# A Whole Germline *BRCA2* Gene Deletion: How to Learn from CNV In Silico Analysis

**DOI:** 10.3390/ijms19040961

**Published:** 2018-03-23

**Authors:** Giovanni Luca Scaglione, Paola Concolino, Maria De Bonis, Elisa De Paolis, Angelo Minucci, Gabriella Ferrandina, Giovanni Scambia, Ettore Capoluongo

**Affiliations:** 1 Fondazione di Ricerca e Cura Giovanni Paolo II, Laboratorio di Oncologia Molecolare, Molipharma a spin-off of Fondazione di Ricerca e Cura Giovanni Paolo II, Contrada Tappino, 86100 Campobasso, Italy; giovanniluca.scaglione@fgps.it (G.L.S.); giovanni.scambia@unicatt.it (G.S.); 2 Istituto Dermopatico dell’Immacolata—Istituto di Ricovero e Cura a Carattere Scientifico, Dipartimento di Diagnostica di Laboratorio e Biologia Molecolare Clinica, 00168 Roma, Italy; 3 Università Cattolica del Sacro Cuore, Fondazione Policlinico Universitario Agostino Gemelli, Polo Scienze delle Immagini, di Laboratorio ed Infettivologiche, 00168 Rome, Italy; paola.concolino@policlinicogemelli.it (P.C.); mari.debonis86@gmail.com (M.D.B.); elisadepaolis88@gmail.com (E.D.P.); angelo.minucci@policlinicogemelli.it (A.M.); 4 Università Cattolica del Sacro Cuore, Fondazione Policlinico Universitario Agostino Gemelli, Polo Scienze della Salute della Donna e del Bambino, 00168 Rome, Italy; mariagabriella.ferrandina@unicatt.it; 5 Laboratory of Molecular Genomics XBiogem, Catholic University of Rome, 00168 Rome, Italy

**Keywords:** *BRCA1/2*, HBOC, NGS, CNV, MLPA, data analysis

## Abstract

*BRCA1/2* screening in Hereditary Breast and Ovarian Syndrome (HBOC) is an essential step for effective patients’ management. Next-Generation Sequencing (NGS) can rapidly provide high throughput and reliable information about the qualitative and quantitative status of tumor-associated genes. Straightforwardly, bioinformatics methods play a key role in molecular diagnostics pipelines. *BRCA1/2* genes were evaluated with our NGS workflow, coupled with Multiplex Amplicon Quantification (MAQ) and Multiplex Ligation-dependent Probe Amplification (MLPA) assays. Variant calling was performed on *Amplicon Suite*, while Copy Number Variant (CNV) prediction by in house and commercial CNV tools, before confirmatory MAQ/MLPA testing. The germline profile of *BRCA* genes revealed a unique HBOC pattern. Although variant calling analysis pinpointed heterozygote and homozygote polymorphisms on *BRCA1* and *BRCA2*, respectively, the CNV predicted by our script suggested two conflicting interpretations: *BRCA1* duplication and/or *BRCA2* deletion. Our commercial software reported a *BRCA1* duplication, in contrast with variant calling results. Finally, the MAQ/MLPA assays assessed a whole *BRCA2* copy loss. In silico CNV analysis is a time and cost-saving procedure to powerfully identify possible Large Rearrangements using robust and efficient NGS pipelines. Our layout shows as bioinformatics algorithms alone cannot completely and correctly identify whole *BRCA1/2* deletions/duplications. In particular, the complete deletion of an entire gene, like in our case, cannot be solved without alternative strategies as MLPA/MAQ. These findings support the crucial role of bioinformatics in deciphering pitfalls within NGS data analysis.

## 1. Introduction

Heterozygous germline mutations in either *BRCA1* or *BRCA2* that impair their normal function confer significantly elevated risks of breast, ovarian, and other cancers [1]. *BRCA1* gene deletions are not frequent, accounting for only 5–10% of all germline mutations and these are probably even less common in *BRCA2* [2]. A wide range of genetic alterations occurring in *BRCA* genes may lead to a truncated or functionless protein, as reported [3] in Breast Cancer Information Core (BIC) database: these are most frequently classified as small deletions or insertions, non-sense mutations and splice variants. Nevertheless, an increasing number of large genomic rearrangements (LGRs), not detectable by current polymerase chain reaction (PCR)-based methods, has been identified in these genes [4]. 

To investigate the *BRCA1/2* germline status of our Hereditary and/or Sporadic Ovarian Cancer women, we have recently validated a massive parallel sequencing (MPS)-based pipeline [5]. The diagnosis of HBOC is made following molecular genetic testing in an individual or family with a germline *BRCA1* or *BRCA2* pathogenic variant [6]. Our molecular diagnostic workflow is based on BRCA MASTR Dx kit by Multiplicom, a In Vitro Diagnostic Medical Device in Europe (CE-IVD) validated kit that is widely used for the genetic screening of whole coding and exon-flanking regions of *BRCA1* and *BRCA2* genes on Illumina platforms. Notably, MPS protocols have increased reliably and rapidly the range of information achievable from these second generation sequencing assays. Consequently, well designed PCR-based multiplex kit coupled with high-throughput bench-top hardware are now leading to a more accurate understanding of both qualitative and quantitative information concerning the germline status of many patients within a single run. We have implemented and internally validated our pipeline on MiSeq (Illumina, San Diego, CA, USA) that is now routinely used in our laboratory to genotype *BRCA* genes in more than two thousand patients per year. MiSeq system is a bench-top instrument that uses Sequencing-by-Synthesis (SBS) approach to generate up to 300 bp pair-end reads by means of solid-phase bridge amplification. The output ranges from 1 to 25 millions of reads per run, accounting for 540 Mega-base (Mb) up to 15 Giga-base (Gb). The DNA of each sample is tagged with specific sequences, or multiplex-identifiers (MIDs), prior pooling, and loading on the sequencer [7,8]. 

Since molecular diagnostic pipeline was specifically designed to guarantee quality control steps and confirmatory testing other than MPS assay throughout the entire workflow, Fragment Analysis (FA) has been early introduced to detect small indels within the PCR amplicons just before the MPS run. Furthermore, a set of in house bioinformatics tools was tailored to drive supplementary quality control and evaluation, encompassing the analysis of digital data that are generated throughout each analytical run. To detect pathological exon copy number changes within both genes among our tested patients, we started using Sophia DDM v3 commercial software. Finally, Sanger sequencing and both Multiplex Amplicon Quantification (MAQ) and Multiplex Ligation-dependent Probe Amplification (MLPA) analysis were performed as confirmatory tests for both variant calling and copy number variants, respectively. MLPA is a multiplex PCR method consisting of two adjacent probes that are linked by ligase when they hybridized on the target sequence. All of the ligated probes share identical sequences at their outermost ends permitting their simultaneous amplification using an unique primers pair. The amplification products are separated by capillary electrophoresis (CE) due to stuffer sequences of different lengths placed at the 3-prime end of one of each target-specific probe [9]. On the other hand, the MAQ method is based on multiplex PCR of several fluorescently labeled target and reference sequences. To detect copy number alterations, the fluorescent signals from the reference and target amplicons, obtained by test and control individuals, are evaluated after the fragment analysis performed on CE system [10,11]. 

To date, Nunziato et al. [12] recently published their single solution to easily address the germline status of a breast cancer patient carrying large *BRCA2* exon 4-26 duplication. Therefore, the predictive algorithm used in their setup was able to match the correct CNV. On the other side, we underline that it is challenging to offer a single solution to address all of the genomic alterations occurring in routine analysis. As a matter of fact, an entire or very large gene duplication is straightforward to detect. In this targeted amplification strategy, where each gene turns into the reference for copy number evaluation of the spare one, this offsets and clarifies the opposite confidence in evaluating a duplication or a deletion of this extent. Nevertheless, we were able to correctly achieve the molecular diagnosis of whole *BRCA2* gene deletion by means of the workflow described in this paper. Although this is an extremely rare scenario, we want to point out that commercial computational tools were not able to address the real copy number status of *BRCA2* gene in the herein reported ovarian cancer case, where the complete deletion of *BRCA2* gene was wrongly predicted as a complete duplication in *BRCA1*. We describe herein the complete laboratory setting of the workflow that is used to decipher and solve this issue.

## 2. Results

### 2.1. Fragment Analysis (FA)

To check the performance of PCR-enriched bar-coded amplicon libraries on the samples amplified using BRCA MASTR Dx kit, five different plexes consisting of 93 amplicon generated on plex A (17 amplicons), plex B (20 amplicons), plex C (19 amplicons), plex D (18 amplicons), and plex E (19 amplicons), were fluorescently labeled and checked for semi-quantitative analysis on 3500 Sequence Analyzer (Appendix A).

**Figure 1:**
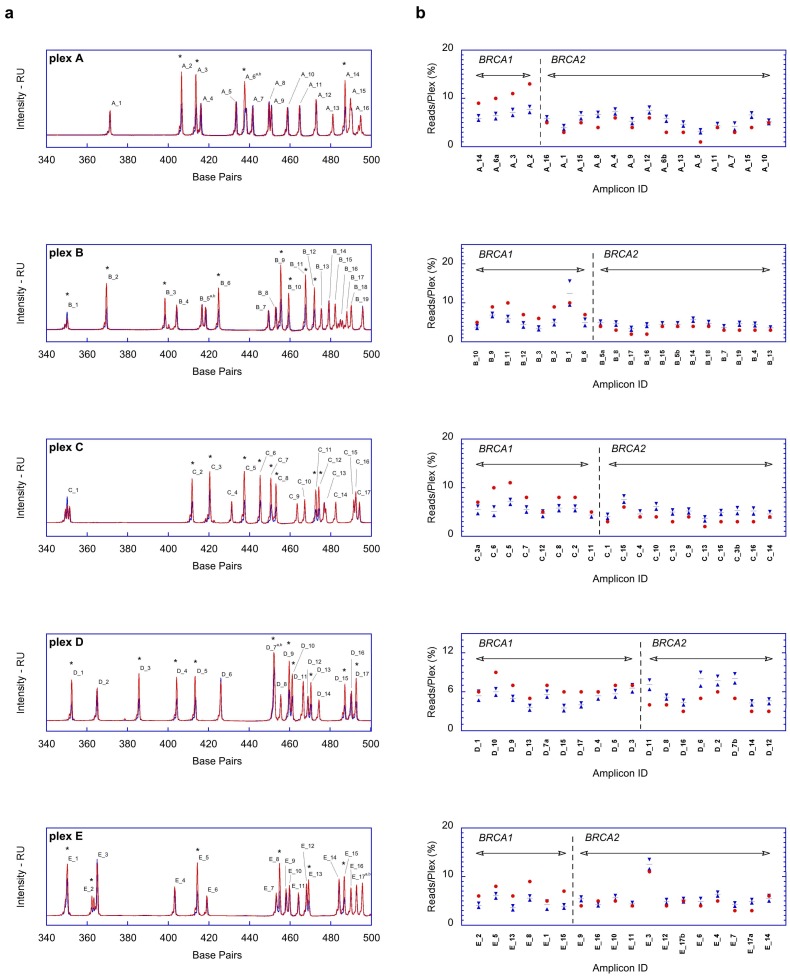
Quality control analyses performed with in house script on fragment analysis (FA) and Next-Generation Sequencing (NGS) dataset. (**a**) Superimposition of normalized electropherograms obtained from the five plexes of the Sample 5, (whole deletion of *BRCA2*, red line) and True Negative sample, True Negative CNV (TN-CNV) (blue line). The plot shows the distribution of the amplicon resolved in each plex, during Fragment Analysis assay, as a function of base pair length by capillary electrophoresis just before NGS data. For each peak the corresponding ID, according to Table A1, is reported. All of the amplicons with altered profile are flagged by an asterisk (**b**) *BRCA1* and *BRCA2* Read Coverage (RC) frequency values in all plexes of Sample 5, showing the relative amount of polymerase chain reaction (PCR) products belonging to *BRCA1* amplicon and *BRCA2* (red circles). The RC average and confidence intervals (CI99%) calculated using the run statistics (plotted as blue dashes and blue triangles, respectively).

Due to the lacking of internal CNV control in the BRCA MASTR Dx assay, the profiles of the amplicons representing both *BRCA1* and *BRCA2* library products were collected and compared with “True Negative CNV” (TN-CNV) dataset previously confirmed by MLPA/MAQ routine (Figure 1a). To compare the electropherograms of each sample with TN-CNV, a curve-fitting algorithm was applied. Basically, a four-step Visual Basic for Applications (VBA) script was designed: (i) To import the electropherogram raw data of the sample, reference and standards for each plex; (ii) to convert the scan into base pairs data, applying a linear fitting to the datasets of the GeneScan 600 LIZ Size Standard (ThermoFisher, Waltham, MA, USA) assayed in the well of both the samples and reference; (iii) to identify and collect out-of-range amplicons, performing the gene normalization on the sample datasets. The peak heights of *BRCA1* amplicons were used to normalize over reference and then the ratios of each *BRCA2* amplicon were evaluated. A similar process was applied using *BRCA2* heights as a reference: the results are then assembled and plotted; (iv) to generate an interactive plot representing sample against reference curves, featured with *x*-*y axis* adjustment controls. In this way, we were able to obtain a fast and fine alignment of each single amplicon in term of height ratios and base pairs distances.

**Figure 2:**
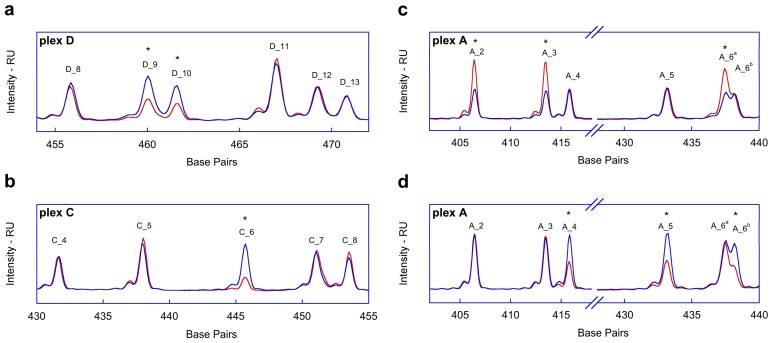
Detailed views of the fragment analysis profiles (red lines) using *in house* script of one sample carrying a deletion of a region of 2664 base pair in *BRCA1*, as an example (**a**,**b**), and the Sample 5 (**c**,**d**) against True Negative-CNV electropherogram profile (blue line). (**a**) Deletion of exon 5 and exon 7 of *BRCA1* within plex D, herein reported with an asterisk and labeled as D_9 and D_10, respectively. (**b**) Deletion of exon 6 on *BRCA1* within plex C, herein reported with an asterisk and labeled as Amplicon C_6. (**c**,**d**) Profiles of a subset of amplicons from plex A, after *gene* normalization on Sample 5 data (red lines): (**c**) normalization of *BRCA2* amplicons peak heights (reference amplicons A_4, A_5, A_6^b^) shows that *BRCA1* peaks are nearly twice those expected (reference pattern, blue line); while (**d**) applying normalization of *BRCA1* amplicon peak heights (amplicons A_2, A_3, A_6^a^), all peaks belonging to *BRCA2* are halved against reference (blue line).

Noteworthy, as shown in Figure 2a,b, a deletion of 2664 base pairs ranging from chr17:41256109 to chr17:41258773 (NC_00017.10, GRCh37.p13, Release 105) carried by a patient with complete deletion of *BRCA1* exon 5, 6, and 7 (NM_007294.3, from c.135-223 to c.441+30) was early detected in the same experimental conditions (run data not reported). On the other hand, taking into account all five plexes, the interpretation of the candidate profile suggested two diametrically opposite conditions: the complete *BRCA1* gene duplication as well as the whole *BRCA2* deletion (Figure 2c,d).

### 2.2. MPS Analysis

The entire coding regions of the *BRCA1* and *BRCA2* genes were sequenced using BRCA MASTR Dx kit on MiSeq platform. MPS data were then analyzed to evaluate *BRCA1* and *BRCA2* amplicon read count (RC) ratios in each plex.

Based on our validated molecular diagnostic pipeline for *BRCA* genes alterations on MiSeq platform, the confidence intervals (CI99%) for each amplicon were already provided and herein applied in data filtering/checking during post-Next-Generation Sequencing (NGS) analysis step (Figure 1b). Read Coverage analysis performed with our scripts reveals that global *BRCA1/BRCA2* ratio in the five plexes of Sample 5 was extremely higher ~1.3 and remarkably out of the range of 0.7 ± 0.2 (Mean ± DS) gathered from the other samples.

Moreover, coupled data from FA and RC analysis clearly suggested the needs of further CNV analysis to assess the real copy number status of *BRCA* genes. Thus, we performed an in silico CNV analysis after MiSeq run by means of commercial DDM bioinformatics tool provided by SOPHiA Genetics (SOPHiA Genetics, Saint-Sulpice, Switzerland). As expected, the CNV prediction data from SOPHiA DDM v3 matched with RC behavior in this sample as well as for the carrier of the deletion of 2664 base pairs, ranging from chr17:41256109 to chr17:41258773 (supplementary data: Figure S1).

### 2.3. MAQ Analysis

**Figure 3:**
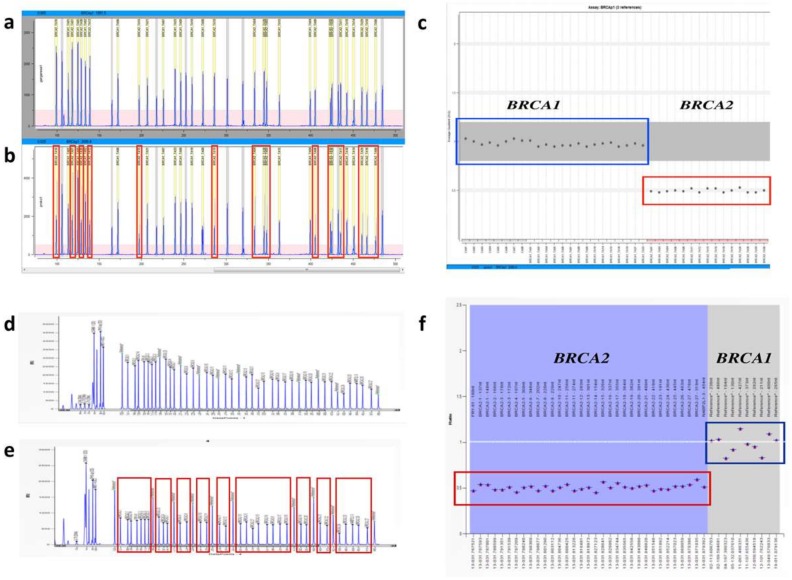
Multiplex Amplicon Quantification (MAQ) (**a**–**c**) and Multiplex Ligation-dependent Probe Amplification (MLPA) (**d**–**f**) analysis results of *BRCA2* gene deletion. (**a**) MAQ electropherogram (Plex 1) from a wild type sample. (**b**) MAQ electropherogram (Plex 1) from the patient carrying whole *BRCA2* deletion, the peaks belonging to *BRCA2* are grouped in red boxes. (**c**) MAQ results by means of Dosage Plot obtained by MAQ-S v2.0 software. It shows an overview of all amplicons and their individual Dosage Quotient indicating the Copy Number. (**d**) MLPA electropherogram from a wild type sample and (**e**) MLPA electropherogram from the patient carrying *BRCA2* deletion where the peaks belonging to *BRCA2* are grouped in red boxes. (**f**) MLPA results: Blue rhombus, black crosses and red triangles indicate, respectively, the maximum, average and minimum values of each probe within the experiment. Probes relative to *BRCA2* show a RPR value of about 0.5 (red box) compared to the normal range value of reference probes (blue box, value: 0.7–1.3), thus indicating (*BRCA2*) exons deletion.

MAQ technique was able to detect *BRCA2* complete deletion (Figure 3a–c). The panel b of the figure reports the electropherogram (Plex 1) of our patient where reduced peak heights of *BRCA2* amplicons were present, as compared to those of wild type reference sample (shown in the Figure 3a). All of the Dosage Quotient (DQ) values were about 0.5 for overall *BRCA2* amplicons targeted by our method, as shown in the final DosPlot analysis, including four reference controls (Figure 3c). Finally, to confirm this result, we also used MLPA analysis, as reported below.

### 2.4. MLPA Analysis

MLPA test confirmed the complete *BRCA2* deletion in our patient (Figure 3d–f). The panel e of the figure shows the patient’s electropherogram with decreased peak heights of all the deleted *BRCA2* exons; the obtained RPR (relative peak ratio) values (normal range 0.7–1.3) were about 0.5 (Figure 3f).

## 3. Discussion

Massive Parallel Sequencing technologies have increased the feasibility of molecular test screening in cancer diagnostics. Furthermore, several studies are ongoing in order to evaluate the benefits that are provided by this technology in routine diagnostics, above all in ovarian cancer *BRCA1/2* assessment [13,14,15,16,17]. NGS platforms are able to provide reliable qualitative data: nevertheless, one of the main challenges regards its ability to precisely identify quantitative changes, like gain or loss in the genes investigated [18,19]. Allele status evaluation is becoming a powerful marker of genome instability, being one of the defected targeted by PARP-1 inhibitors. Therefore, both qualitative and quantitative complete *BRCA1/2* NGS evaluation should be assessed to identify patients who can benefit from PARP-1 inhibitors treatment [20,21]. The strategies are able to detect larger deletions or duplications that may not directly translate from germline DNA to FFPE-derived DNA as consequence of several factors. Important features reflecting the limitations of tumor testing are mainly associated to smaller DNA fragment size, chemical modifications and chromosomal copy number changes, like aneuploidy, and the frequent instability of tumor samples. Consequently, these approaches can be really critical to achieve when *BRCA1/2* testing is performed on both FFPE and fresh tumor level [17].

As a consequence, several papers have been published regarding the identification of large rearrangements by applying different algorithms to the sequencing output data from HiSeq/MiSeq (Illumina) and GS 454 (Roche, recently discontinued) platforms as well as PGM/Ion Proton (Ion Torrent Invitrogen) instruments [22,23,24,25,26,27].

One of the main concerns regarding the bioinformatics tools is their ability to precisely predict copy number status, above all when a large alteration or rearrangement is present: these issues and pitfalls can be relatively more critical depending on type of starting materials (FFPE, rather than fresh or germline deriving DNAs).

In order to show one of the possible pitfalls occurring by using CNV bioinformatics prediction tools in the evaluation of *BRCA1/2* allele status, we report herein an extremely rare case that is characterized by a complete deletion of *BRCA2* genes found in our laboratory. At the beginning, our patient (namely, Sample 5) undergone QC test to verify the behavior of multiplex amplification products according to Multiplicom kit procedure. Consequently, we exported curve data and launched the VBA script to compared unknown sample against a pool of samples previously verified as being normal for *BRCA1/2* copy number (TN-CNV). Therefore, during the analysis we were impressed due to atypical pattern that was contemporarily dealing with both *BRCA1* duplication (Figure 2c) and *BRCA2* deletion (Figure 2d). The subsequent dataset that was obtained on MiSeq machine did not show any pathogenic variants on both genes: nevertheless, such *BRCA1* polymorphisms were in heterozygous and/or homozygous, while 100% of *BRCA2* variants resulted only in a homozygote status. Therefore, we evaluated the total read coverage of both *BRCA1* and *BRCA2* genes comparing these data with those obtained on the other samples processed within the same run. Clearly, *BRCA1* amplicons appeared as overrepresented with an overall *BRCA1/BRCA2* ratio (~1.3), which was twice when compared to that shown by the other samples. In order to decipher this feature, Sophia DDM software was used: unfortunately, the latter was still unable to correctly define CNV status on Sample 5 and the result obtain was inconclusive. We underline as the identification of small rearrangements was easily achieved with both BRCA MASTR Dx and Sophia DDM algorithms on other samples that were previously processed by our group. Not surprisingly, by means of MLPA and MAQ tools, we were not able to confirm the prediction given by Sophia DDM software. By contrast, we report herein the first case of entire *BRCA2* gene deletion that is unique and rare in HBOC syndrome. A similar finding was recently published by Purshouse et al. [28] in the context of WGS analysis on prostate cancer tumor samples, where the whole *BRCA2* deletion was exclusively somatic. Indeed, the present case regards the germline complete *BRCA2* deletion occurring in a woman suffering from ovarian cancer in the context of HBOC syndrome. 

## 4. Materials and Methods 

### 4.1. DNA Samples

All of the subjects gave their informed consent for inclusion before they participated in the study. The study was conducted in accordance with the Declaration of Helsinki, and the protocol (Protocol ID: 0007205/16) was approved by the Ethics Committee of Università Cattolica del Sacro Cuore, Fondazione Policlinico Universitario Agostino Gemelli (Project ID: ESR14-10185, Approval date: 24/02/2016). Particularly, the patients carrying the whole *BRCA2* deletion was 48 years women from Mediterranean Europe diagnosed with high grade serous ovarian carcinoma. 

Genomic DNA was isolated from peripheral blood samples using High Pure PCR Template Preparation Kits (Roche Diagnostic, Indianapolis, IN, USA), eluted in 100 µL of Elution Buffer (Roche Diagnostic, Indianapolis, IN, USA), quantified by spectrophotometer at 260 nm, and stored at −20 °C until use. Only DNA meeting following requirements: OD260/280 ratio ≥1.7, concentration ≥15 ng/µL, no degradation signals visible on agarose gel, were used in the study. 

### 4.2. MPS Analysis

The full coding sequences of *BRCA1* and *BRCA2* genes were PCR-enriched using the BRCA MASTR Dx v2.0 assay (Multiplicom, Niel, Belgium), according to the manufacturer’s instructions. This diagnostic CE-IVD kit is fully compatible with reversible-terminator sequencing technology. Thus, the samples were assayed by means of library pipelines and ran on MiSeq (Illumina, San Diego, CA, USA) platform following our molecular diagnostic routine validated setting [5,20,21,29].

Before MPS run, fragment analysis (FA) was performed on Applied Biosystems 3500 Genetic Analyzer (Life Technologies, Carlsbad, CA, USA) as a Quality Control (QC) to test the enrichment efficacy, the amplicon elongation range, and the amount of primer dimers.

After MPS, the sequencing fastq.gz files from MiSeq were analyzed with a novel CE-IVD bioinformatics tool, namely Amplicon Suite (SmartSeq srl, Novara, Italy), to safely and rapidly investigate variant call (VC) and read count (RC) metrics of sequenced amplicons through its java-based client-server sftp service.

Two different in house scripts were also developed and applied: (i) To collect and analyze the FA profile of the five plexes concerning assayed bar-coded libraries. The extra files were generated and exported from Multiplex Amplicon Quantification (MAQ) software during amplicon library QC analysis. (ii) to deeply investigate read coverage data and PCR stoichiometry of the 93 amplicons belonging to each sample after each MiSeq run. The abovementioned scripts were early programmed in Visual Basic for Application 7 in Microsoft Office and they were completely rewritten and validated in a command line Unix environment by using a combination of bash, Python, Perl, and R software packages to fit to our analytical workflow requirements.

Copy Number Variant (CNV) analyses were also performed in silico on MiSeq dataset by means of commercial software Sophia DDM v3 (Sophia Genetics SA, Saint Sulpice, Switzerland).

### 4.3. MAQ Analysis

The MAQ v1.0 kit (Multiplicom, Niel, Belgium) was used according to reported instructions. Briefly, after DNA quantification, two steps were performed: PCR reaction and fragment analysis. In the first step, two multiplex PCR reactions were prepared for each patient: 20–50 ng of DNA were used in a final reaction volume of 15 µL, including 5 µL of Master reaction mix (Plex A or Plex B) and sterile distilled water. After 10 min to 98 °C, 23 cycles (95 °C 45 s, 60 °C 45 s and 68 °C 2 min) were performed with a final step to 72 °C for 10 min. Regarding fragment analysis, 2 µL of the MAQ PCR product was added to a well containing the size standard GS600 (Applied Biosystems, Warrington, UK) (0.3 µL) and 10 µL of HiDi-Formamide (Applied Biosystems, Warrington, UK). The run was performed on 3500 Genetic Analyser (Applied Biosystems, Warrington, UK) and MAQ-S v2.0 analysis software (Multiplicom, Niel, Belgium) was used for analysis results. Four healthy individuals were included in the analysis as reference controls.

### 4.4. MLPA Analysis

The SALSA MLPA kits for *BRCA1* (P002-D1; MRC-Holland, Amsterdam, The Netherlands) and *BRCA2* (P090-A4; MRC-Holland, Amsterdam, The Netherlands) were used for the relative quantification of all *BRCA1/2* exons according to the manufacturer’s instructions. Briefly, 100 ng of genomic DNA were denatured at 98 °C and hybridized with the specific MLPA probe mix at 60 °C overnight. After ligation reaction of annealed probes (54 °C for 15 min), the subsequent PCR reaction was performed for 35 cycles (30 s at 95 °C, 30 s at 60 °C, 60 s at 72 °C), with a final step at 72 °C for 20 min. 0.7 μL of amplification product were then mixed with 0.4 μL of the GS-600 Size Standard (Applied Biosystems, Warrington, UK), 10 μL of HiDi-Formamide (Applied Biosystems, Warrington, UK) and analyzed using an 3500 Genetic Analyzer (Applied Biosystems, Warrington, UK). The collected data were analyzed using Coffalyser.NET Software (MRC Holland, Amsterdam, The Netherlands). Three healthy males and three healthy females were included in the analysis as wild type controls.

## 5. Conclusions

**Figure 4:**
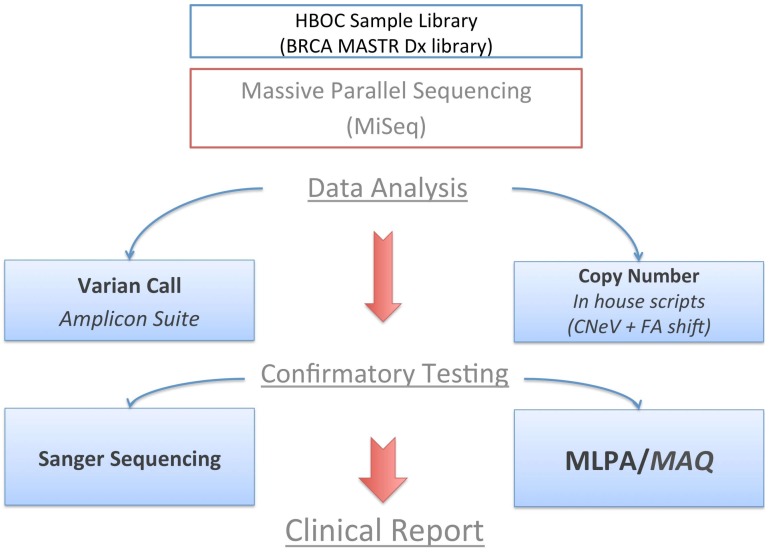
The Standard Operative Procedure (SOP) reflecting the diagnostics workflow used for the identification of the germline *BRCA2* deletion is reported.

We underline how the bioinformatics algorithms could be very powerful and useful in providing quantitative information regarding possible large rearrangements, above all when robust and well-designed NGS pipelines are set up. Nevertheless, PCR-based NGS assays cannot still correctly predict CNVs, with this task being challenging. Moreover, in silico analysis coupled with MPS platforms could be suitable for both qualitative and quantitative gene profiling (Figure 4).

Herein, we emphasize as the risk of misinterpretation of patient’s CNV status, performed by means of in silico tools, can be overcome through the deep knowledge of: (a) NGS chemistry biases; (b) complete pipeline design; and, (c) amplicon dynamics and behaviors within any run. 

Our algorithm met this need since our pipeline was tailored using results obtained on thousands of samples consecutively processed with our validated diagnostic workflow: therefore, the pattern of any amplicon amplified within any plex was deeply characterized. This strategy could result as very promising for the identification pitfalls regarding copy number status assessment. This is the reason why laboratories providing *BRCA*1/2 NGS-based assays, should guarantee complete qualitative and quantitative analysis of both genes, in order to overcome these issues and facilitate patients’ management.

## Supplementary Materials

Supplementary materials can be found at http://www.mdpi.com/1422-0067/19/4/961/s1.

## Abbreviations

*BRCA1/2*	BReast CAncer susceptibility genes 1/2
CNV	Copy Number Variant
DDM	Data-Driven Medicine
FFPE	Formalin-Fixed Paraffin-Embedded
HBOC	Hereditary Breast and Ovarian Syndrome
MLPA	Multiplex Ligation-dependent Probe Amplification
MAQ	Multiplex Amplicon Quantification
MPS	Massively Parallel Sequencing
NGS	Next Generation Sequencing
PGM	Personal Genome Medicine
VBA	Visual Basic for Applications
WGS	Whole Genome Sequencing

## Appendix A

**Table A1:** Details regarding amplicons generated by BRCA MASTR Dx pipeline.

Plex A		Plex B		Plex C		Plex D		Plex E	
ID	Amplicon	ID	Amplicon	ID	Amplicon	ID	Amplicon	ID	Amplicon
A_1	BRCA2_ex09_01	B_1	BRCA1_ex20_01	C_1	BRCA2_ex06_01	D_1	BRCA1_ex03_01	E_1	BRCA1_ex22_01
A_2	BRCA1_ex23_01	B_2	BRCA1_ex19_01	C_2	BRCA1_ex14_01	D_2	BRCA2_ex11_13	E_2	BRCA1_ex09_01
A_3	BRCA1_ex11_12	B_3	BRCA1_ex16_02	C_3a	BRCA1_ex02_01	D_3	BRCA1_ex17_01	E_3	BRCA2_ex11_14
A_4	BRCA2_ex11_07	B_4	BRCA2_ex25_02	C_3b	BRCA2_ex19_01	D_4	BRCA1_ex13_01	E_4	BRCA2_ex21_01
A_5	BRCA2_ex13_01	B_5a	BRCA2_ex02_01	C_4	BRCA2_ex10_04	D_5	BRCA1_ex16_01	E_5	BRCA1_ex10_01
A_6a	BRCA1_ex11_07	B_5b	BRCA2_ex11_09	C_5	BRCA1_ex11_05	D_6	BRCA2_ex11_11	E_6	BRCA2_ex12_01
A_6b	BRCA2_ex11_19	B_6	BRCA1_ex21_01	C_6	BRCA1_ex06_01	D_7a	BRCA1_ex11_06	E_7	BRCA2_ex22_01
A_7	BRCA2_ex15_01	B_7	BRCA2_ex16_01	C_7	BRCA1_ex11_08	D_7b	BRCA2_ex14_02	E_8	BRCA1_ex11_10
A_8	BRCA2_ex11_01	B_8	BRCA2_ex08_01	C_8	BRCA1_ex12_01	D_8	BRCA2_ex10_03	E_9	BRCA2_ex03_02
A_9	BRCA2_ex11_10	B_9	BRCA1_ex11_01	C_9	BRCA2_ex11_12	D_9	BRCA1_ex05_01	E_10	BRCA2_ex11_02
A_10	BRCA2_ex24_01	B_10	BRCA1_ex08_01	C_10	BRCA2_ex11_05	D_10	BRCA1_ex07_01	E_11	BRCA2_ex11_06
A_11	BRCA2_ex14_01	B_11	BRCA1_ex11_14	C_11	BRCA1_ex18_01	D_11	BRCA2_ex05_01	E_12	BRCA2_ex11_17
A_12	BRCA2_ex11_15	B_12	BRCA1_ex15_01	C_12	BRCA1_ex11_13	D_12	BRCA2_ex27_03	E_13	BRCA1_ex11_02
A_13	BRCA2_ex11_21	B_13	BRCA2_ex27_02	C_13a	BRCA2_ex11_08	D_13	BRCA1_ex11_03	E_14	BRCA2_ex27_01
A_14	BRCA1_ex11_04	B_14	BRCA2_ex11_18	C_13b	BRCA2_ex11_16	D_14	BRCA2_ex25_01	E_15	BRCA1_ex24_01
A_15a	BRCA2_ex10_01	B_15	BRCA2_ex11_04	C_14	BRCA2_ex23_01	D_15	BRCA1_ex11_09	E_16	BRCA2_ex04_01
A_15b	BRCA2_ex18_01	B_16	BRCA2_ex10_05	C_15a	BRCA2_ex18_02	D_16	BRCA2_ex11_03	E_17a	BRCA2_ex11_22
A_16	BRCA2_ex03_01	B_17	BRCA2_ex10_02	C_15b	BRCA2_ex07_01	D_17	BRCA1_ex11_11	E_17b	BRCA2_ex26_01
		B_18	BRCA2_ex11_20	C_16	BRCA2_ex20_01				
		B_19	BRCA2_ex17_01						

The codes of amplicons pooled in each plex were shortened. Reference pattern from Multiplicom documentation: BRCA MASTR^TM^ GeneScan Profiles file is herein reported as a guide for amplicon identification.
