# Supplementary material for: A Whole Germline BRCA2 Gene Deletion: How to Learn from CNV In Silico Analysis
Source: Int J Mol Sci. 2018 Mar 23;19(4):961. doi: 10.3390/ijms19040961 (PMC5979302; doi:10.3390/ijms19040961)
Supplement: Supplementary file 1 [file ijms-19-00961-s001.pdf]

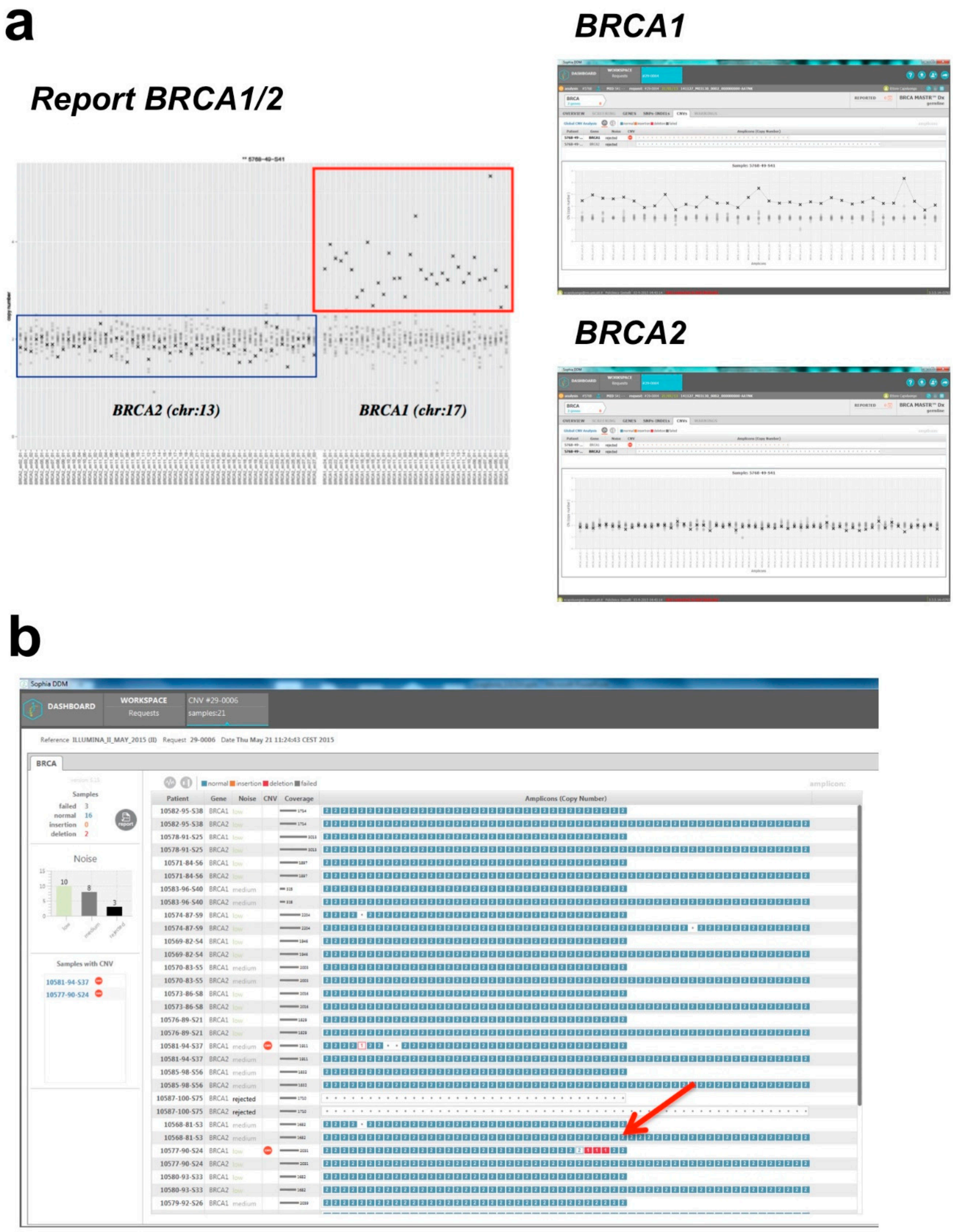

Figure S1. *In silico* CNV analysis with Sophia DDM performed on MiSeq run dataset on V3 chip. **(A)** The raw data of the CNV prediction of the *Sample 5*, carrying whole germline *BRCA2* deletion, is reported on the left. The details of CNV estimations for *BRCA1* and *BRCA2* are represented on the right. **(B)** The sample carrying the deletion of *BRCA1* exons 5-7 is indicated by the red arrow.
